# Feedback promotes learning success! Which kind of feedback for the faculty is given by an interdisciplinary OSCE with focus on decision-making?

**DOI:** 10.3205/zma001052

**Published:** 2016-08-15

**Authors:** Tina Stibane, Helmut Sitter, Despina Neuhof, Helena Wiechens, Andrea Schönbauer, Stefan Bösner, Erika Baum

**Affiliations:** 1 Universität Marburg, Fachbereich Medizin, Dr. Reinfried Pohl-Zentrum für medizinische Lehre (RPZ), Marburg, Germany; 2 Universität Marburg, Dekanat Medizin, Marburg, Germany; 3 Praxis für Allgemeinmedizin, Braunfels, Germany; 4 ehem. Universität Marburg, Fachbereich Medizin, Dr. Reinfried Pohl-Zentrum für medizinische Lehre (RPZ), Marburg, Germany; 5 Universität Marburg, Abteilung für Allgemeinmedizin, Präventive und Rehabilitative Medizin, Marburg, Germany

**Keywords:** medical education, competency-based assessment, feedback-role of assessment, faculty development

## Abstract

Clinical skills such as history taking, diagnostic reasoning, therapy planning, and giving advice are even more complex than practical skills like lung auscultation and have to be applied in complex clinical situations. We judged this competence in an interdisciplinary formative OSCE conducted with students of Marburg University. Results of 218 students passing 643 OSCE stations composed of 37 different scenarios were analyzed. As a competence based examination that reflects the practical skills gained during clinical training, the here presented analysis serves also as a feedback instrument for clinical teachers, their respective disciplines and the medical faculty as a whole.

## Zusammenfassung

Praktische klinische Fertigkeiten, die klinisches Denken erfordern und über technisches Können hinausgehen, lassen sich schwer prüfen. Anamneseerhebung, zielgerichtetes diagnostisches Vorgehen, differentialdiagnostisches Denken, Therapieempfehlung und Beratung müssen in verschiedensten komplexen Anforderungssituationen angewendet werden und sind selber komplexer als isolierte Aufgaben wie Lungenauskultation. Bei Marburger Studierenden der Humanmedizin im 3. klinischen Studienjahr wird diese Kompetenz in einem fächerübergreifenden und formativen OSCE-„Entscheidungsfindung“ überprüft. Die Leistungen von 218 Studierenden im Studienjahr 2014/2015 wurden statistisch ausgewertet. Die Analyse der Ergebnisse gibt den beteiligten Lehrenden, ihren Fächern und auch der Fakultät als Ganzes ein Feedback über ihre Lehre.

## 1. Introduction

During the last years objective structured clinical examinations (OSCEs) are widely used in medical education in Germany [1]. They are well suited to test practical skills of students [2], [3], [4]. With OSCE at Philipps-University Marburg it was shown, that basic practical skills of physical examination could be improved by structured practical teaching [5]. The ability for a target oriented clinical examination, which students have to develop during the course of their studies, is much more complex: the performance of clinical examinations is accompanied by a comprehensive knowledge about diseases and diagnostic criteria. The doctor-patient-interaction is more complex, if it is not only a modular task for a test for students. During students’ education decision making is more and more in the focus and discussed in clinical courses. Explicitly this is the case in the integrated lecture of the third clinical year and in the seminar general practice, which accompanies the corresponding clinical course. This is the reason for establishing an interdisciplinary assessment in the curriculum of Marburg, which tests different competences of physicians: the “OSCE decision making”. Within the frame of two dissertations (Neuhof D, Wiechens H) and in cooperation with the department of general practice and a centre of medical education, belonging to the deanery of education, case vignettes, scripts for role plays, and evaluation criteria were developed, tested, and identified as suitable. For the choice of case vignettes, which had to be dealt with at the different stations, the frequency and relevance of different causes for consultations in the primary care setting were relevant. At the same time it should be possible to test a broad range of basic practical skills with the case vignettes. Especially their matching with practical skills in the curriculum of the department and with the National Competence Based Catalogues of Learning Objectives for Undergraduate Medical Education (NKLM) [6] was emphasized. Designed as an interdisciplinary examination in a setting of a general physician, during the course of time more and more clinical disciplines were motivated for participating at OSCE-decision making.

In this way a pool of over 40 stations was generated, and every student in the third clinical year visits - according to a random principle - three stations with 20 minutes each, directly after the practical course “general practice”. During the first 15 minutes there is interaction between the “physician” (student) and the “patient” (standardised patient), during the last five minutes differential diagnoses and preventabale dangerous courses of disease are discussed. The reviewers give feedback on action, behaviour, and clinical reasoning of the students. During the last five minutes the standardised patients score according to a check list the behaviour of the students with respect to performance, shown empathy, and presentation of information during the interaction with an overall score each. After the examination the students have the chance to know their score by the examiner as well as the score of the interaction in written form as a scholar mark from 1 (very good) to 5 (unsatisfactory). Nevertheless this examination has no relevance for the students’ certificates. The examiners as well as the standardised patients are trained for applying this scoring and the majority of them is experienced. As feedback to the examiners the students could give a comment on the format of the examination with free text at the end. 

An examination which tests the presence of a practical skill which was taught in lessons before, directs the learning behaviour of the students and leads to practical exercise of students and acquirement of job specific and practice oriented competence [5]. The examination of complex decision making which consists of parts from the whole course of the study does not have – as a one shot event during the study - a directive function for learning, because focused rehearsal is not possible. This is one of the reasons why the OSCE - decision making was introduced as a formative examination without any danger of failing. Therefore an otherwise important aspect of an examination, the selection, is missing. Nevertheless this examination should fulfill a feedback function, where the trained standardised patients give additional feedback. Between January 2012 and March 2013 a total of 937 free text answers (cues, lists, whole sentences) from 272 participants from 11 OSCE examinations were evaluated. More than two third (70.43%) of all statements were categorised as positive feedback. Special emphasis is on an additional 1.28% of statements, which recurred on the format of the examination as a chance for learning: “Such examinations are good for self reflected learning! Unfortunately, this shows clearly the problem of theoretical studying. One is forced to think much more in terms of differential diagnoses, for this reason such examinations are great and should be implemented more often.” That this examination provides very good feedback to students was shown by the evaluation of the free texts [7]. However which kind of feedback is generated by this examination format for the involved medical disciplines? Is it possible to recognize the specific positive and negative aspects of the education as a whole and in different clinical disciplines through the results of the students in the OSCE decision making? 

## 2. Description of the project

Every parcours consists of a minimum of 6 and maximum of 10 stations for 14 to 30 students, who take the examination on one date. Every student passes three stations in well-equipped simulation rooms (home visit scenario and private practice), simulator technique is used in addition to the trained standardised patient. Every station is passed by 6 to 9 students. The students are informed about the interdisciplinary formative examination in general at the beginning of their general practice course and about the details and the procedure directly before the examination. At the time of the examination their practical course in private practices is just finished which entails that they are prepared for the setting of the cases of the examination. In contrast to the OSCE of the first clinical year, where a standard procedure for clinical examination techniques was developed, the selected situations here allow different possibilities to achieve the goal. A series of anamnestic questions, physical examinations and other diagnostic actions are necessary as standards in the given setting due to guidelines for general practicioners and expert opinions, but some students relatively quickly get a target-oriented diagnosis in their mind and therefore ask and examine in a clear direction, while others do not, but arrive as well at the right diagnosis. Some steps of a clinical examination are adequate, but not always necessary. Searching techniques for clarifying differential diagnoses belong to this. This is important for the evaluation criteria. A checklist with items, often used in OSCE for practical skills, does not take into account the variance, therefore on the evaluation sheet relevant items of the case are given which include the exclusion of differential diagnoses because of clinical reasoning. However five permanent evaluation criteria were determined for every single step of the interaction and of the differential diagnostic procedure in the course of the development process and with their case specific associated items global scores were introduced. For the scores 1, 3, and 5 requirements are described, the scores 2, and 4 can be used for further differentiation. Evaluated items are (partly word by word) [7]:

1. History taking. In order to avoid the definition of single questions a question-category is built with every case. “Pain” for example, consists completely of all questions about pain, i.e. character of pain, first appearance, course, cause etc. “Diseases and drugs” consists of all important questions for the main symptoms about accompanying, previous, and family diseases and drugs taken.
2. Target-oriented selection of physical examinations and target-oriented administration of diagnostic actions. For each case there is a list of necessary findings, which are on the check list of the examiner. Evaluated is which physical examinations and requests for medical findings are ordered by the student.
3. Physical examination and interpretation of findings. Evaluated are the procedural quality of the performed investigations on the standardised patient or the simulator and the interpretation of the other findings present from the previous requested clinical examination.
4. Proposal of therapy, consulting and termination of interaction. The termination of each case has to make sense, there exist big differences: According to the case the proposal of therapy should be correct, risks for the patient or the future actions should be addressed.
5. Differential diagnostic weighing. The consideration of “preventable adverse courses” as well as the correct diagnosis, which is given on the checklist lead to the score.

### 2.1. Data analysis

The performance of all students, who participated during winter term 2014/2015 and during summer term 2015 at the OSCE decision making was analysed in the criteria “history taking”, “target-oriented selection of physical examinations and target-oriented administration of diagnostic actions”, “quality of taking findings”, “termination of interaction and future actions”, and “differential diagnostic thinking” with respect to distribution of scores, average scores and standard deviation at the different stations as well as the comparison of means at all stations and all disciplines by ANOVA and Scheffe-test in order to test the influence of differences in performance statistically. The significance level was chosen as 0.05. The results of the students at different stations were treated as independent data, because data analysis with dependent data was not necessary for the addressed questions.

The evaluation, which the standardised patients gave on the competence of communication and behavior were analysed, according to their distribution in the categories “performance”, “empathy”, and “information” for all students and stations.

**Figure 1:**
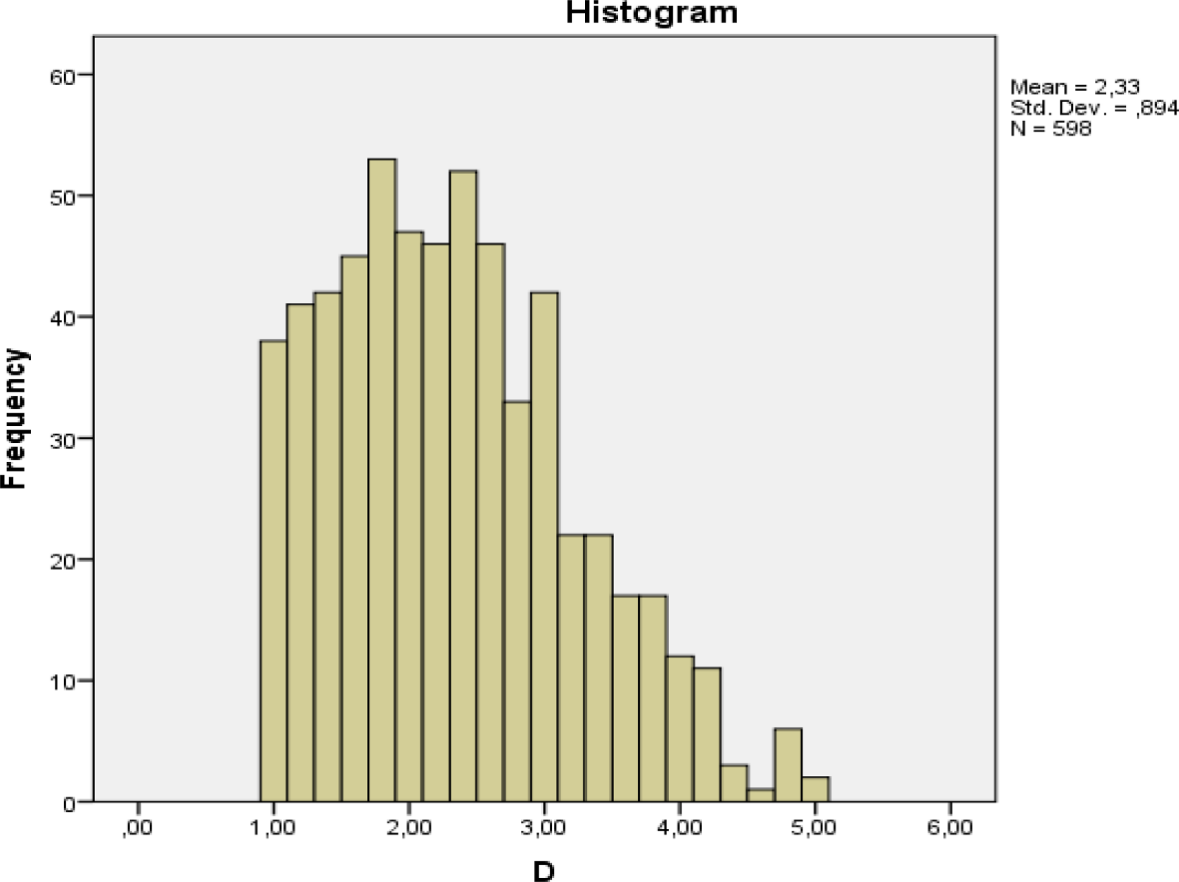
Frequency of mean scores (D) for all students and stations

**Figure 2:**
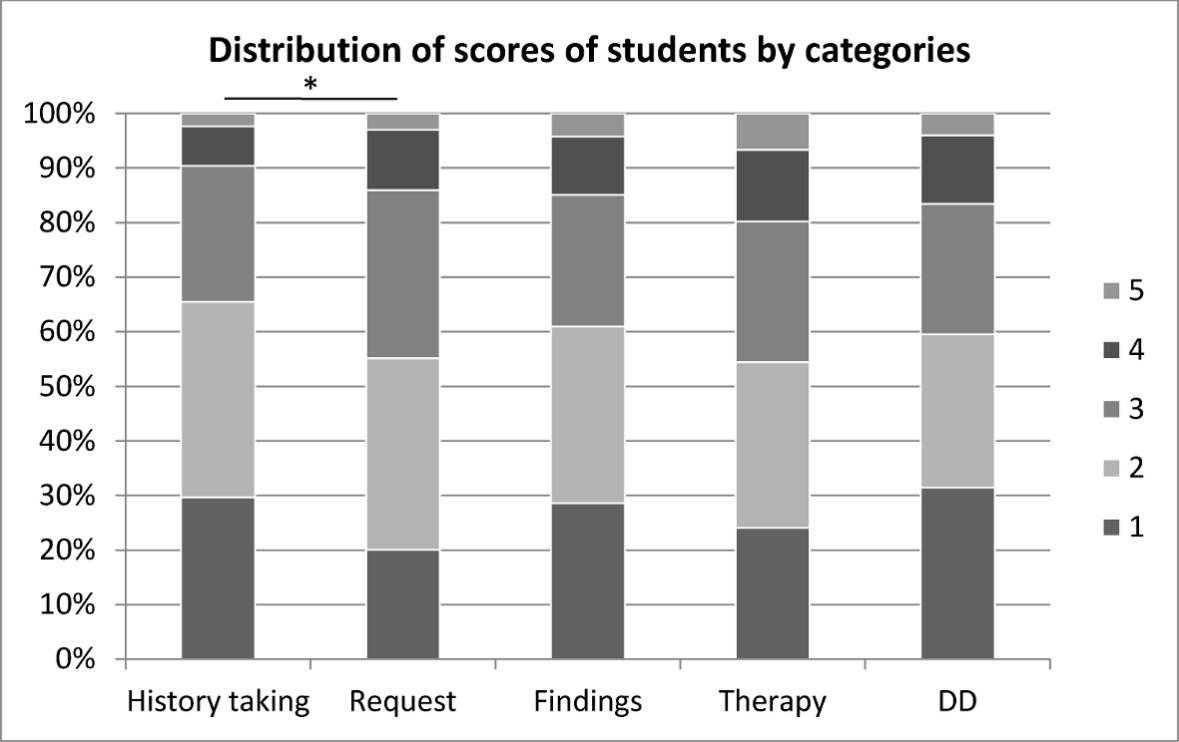
Distribution of scores (1=very good – 5=unsatisfactory) in percent in the five categories history taking, request, findings, therapy, and differential diagnosis (DD)

**Figure 3:**
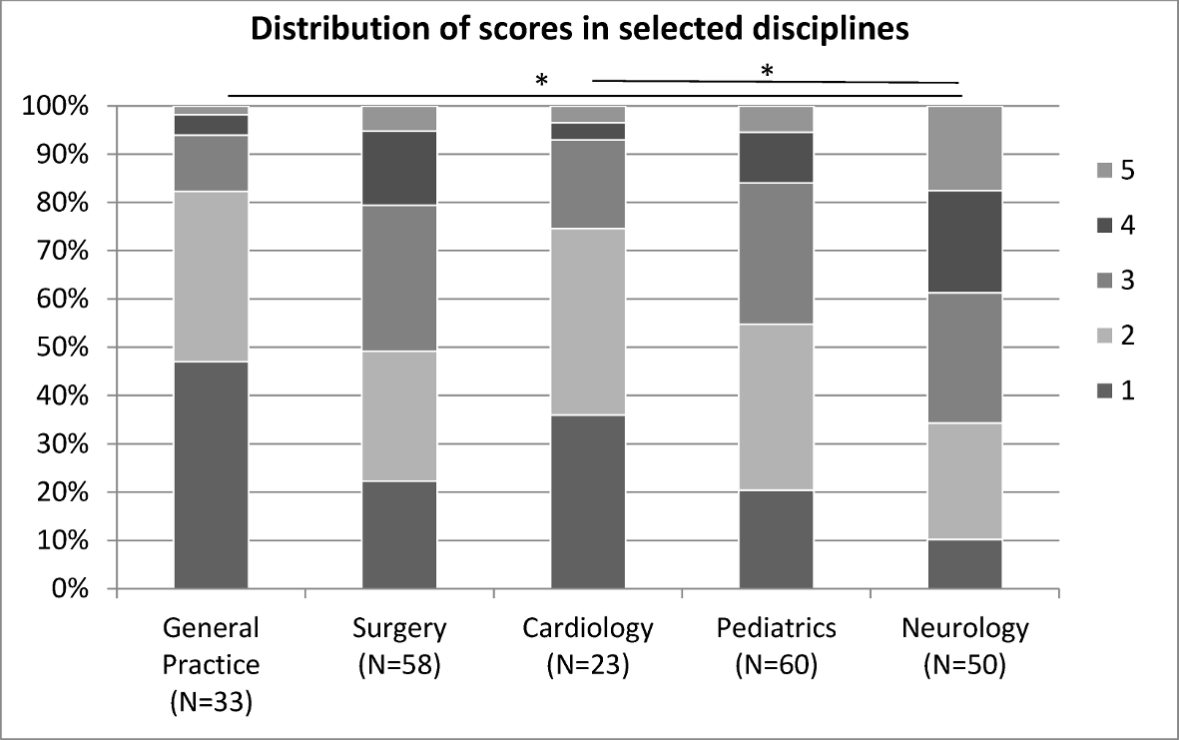
Distribution of mean scores (1=very good – 5=unsatisfactory) in selected disciplines

**Figure 4:**
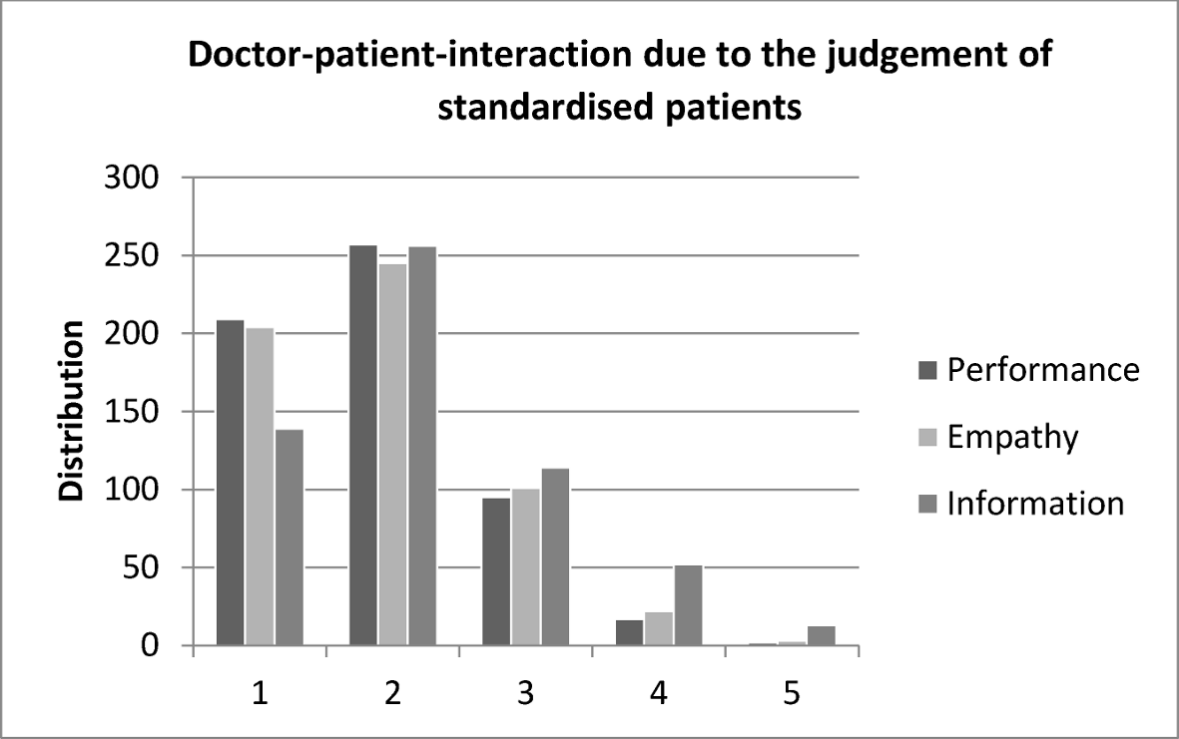
Distribution of scores for doctor-patient-interaction in the categories performance, empathy and information (1=very good - 5=unsatisfactory) due to the judgement of standardised patients

The data analysis (figure 1 (Fig. 1), table 1 (Tab. 1), frequencies, means, standard deviations, significances, ANOVA)) was performed with the statistics software SPSS (IBM SPSS Statistics 22) and for the figure 2 (Fig. 2), figure 3 (Fig. 3) and figure 4 (Fig. 4) the software EXCEL (Microsoft Office Excel 2007) was used. 

## 3. Results

Between November 2014 and July 2015 altogether 218 students who had just finished their practical course general practice participated in an interdisciplinary OSCE decision making. 208 students attended three stations each (altogether 624 stations), 9 students attended only two stations (examiner was late) (altogether 18 stations). One student cancelled the examination due to illness after one station. During this period of time 37 different stations were used, these stations are mapped to 18 clinical disciplines (see table 1 (Tab. 1)).

3160 evaluations were documented on the evaluation sheets of the examiners in the categories history taking, request, findings, therapy and differential diagnosis. Considering all stations 55 single scores (1.71%) are missing (see table 2 (Tab. 2)).

The frequency distribution of the average scores in figure 1 (Fig. 1) has a mean of 2.33 with a left handed skewnees for better scores.

The distribution of the mean values of the scores range for the five categories from a minimum value of 2.17 for history taking as best result to a maximum value of 2.48 for recommendation of therapy as worst result, shown in table 2 (Tab. 2) and figure 2 (Fig. 2). The number of the scores 4 and 5 was only 61 (9.5%) for history taking, but 125 (19.4%) for therapy. For physical examination and interpretation of results the students got in 61% (N=388) of cases the score 1 or 2, but in 15% (N=95) of cases the score 4 or 5. Looking at the distribution of scores only by comparison of means, the post-hoc-test resulted in a significant difference between the categories history taking and request.

Considering the distribution of scores in the disciplines, descriptively there are marked differences, which are significant in the comparison of means by ANOVA, but if every discipline is tested against every other discipline by post-hoc test (Scheffe) the overall difference rests only on a few significant differences between two disciplines. Statistically significant differences of the scores between the five disciplines selected here for illustration purposes are only seen between general practice and neurology as well as between cardiology and neurology. All other differences are statistically not significant (see figure 3 (Fig. 3)).

The standardised patients evaluated for 643 stations 590 valid evaluation sheets with altogether 1729 evaluations of the interaction “doctor” and “patient” (53 times there was no filled in evaluation sheet by the standardised patient, i.e. because of lack of time). The score is missing or equivocal 10 times in the category performance, 15 times in the category empathy, and 16 times in the category information (missing values altogether: 2.31%). Figure 4 (Fig. 4) shows that the standardised patients evaluate the students much more often than average with the scholar marks 1 or 2 (80.34%) and only in 3.2% of the evaluations with 4 or 5. The situation is similar with empathy, 78.08% receive 1 or 2 and only 4.43% receive 4 or 5. The evaluation of “information” was worse, with two third of the evaluations (68.84%) 1 or 2, but also 11.32% of evaluations with 4 or 5. In 10 evaluation sheets students were rated in all three categories with 4 or worse.

## 4. Discussion

The students get an individual feedback on their observed clinical competence by the patient cases from different clinical disciplines, due to the systematics of the stations, and due to the post-examination discussion with the examiner. The performance of the totality of students however expresses how much the students are prepared for the whole spectrum of practical skills of their future job by the teaching of reference disciplines. The average results of all stations in the categories history taking, decision for request of clinical examinations and investigations, termination of “doctor-patient” interaction, and therapy, as well as differential diagnostic feedback give information about the whole teaching process. The reliability of the evaluation was proven to be sufficient in a previous test [Neuhof D], whereas a formal evaluation of validity was not performed. The overall result was, that the students showed a good level of education before the start of the “practical year”. This does not apply to everybody to the same amount: It is apparent, that with positive overall performance, a small part of students (3.6%) at one station in all five categories is inferior to 4.0 and 2 students (<1%) are at all stations inferior to 4.0. In addition the standardised patient´s state marked shortcomings in 10 cases of all evaluated aspects of doctor-patient-communication.

The fact that history taking is part of the curriculum in clinical as well as in communicative teaching in several lectures, resembles the good results for this part of the task at the stations. Here the task to recommend a therapy or risk reducing behaviour to the patient or to explain necessary actions gets the most inferior marks. One reason for this might be, that in this step of the examination a decision about an intervention is warranted and this is addressed in the teaching not in detail, one trusts that this area is taught in detail during the “practical year”.

The difference in the performance of the students according to disciplines can be recognised descriptively, but significant differences in the comparison of the means are present only in a few cases. Consideration of the distribution of scores at a single station give each discipline a chance for detailed reflexion about the causes of evaluation scores and for adaptation of their teaching contents.

The evaluations of the doctor-patient-communication by the standardised patients are very favourable; nevertheless the standardised patients also identify single students, by whom they feel treated not adequately in several aspects. 

The results in total show a positive image of the performance of the students. Furthermore the OSCE decision making as a competence oriented examination is not only well suited as feedback for students but also for the reference disciplines, to check for theoretical teaching content and how it can be applied in a reality-near situation. In addition the OSCE decision making can be used as orientation by the faculty to decide, whether the education of students is sufficient successful with respect to measurable competences in the examination.

A shortcoming might be that students perhaps take a formative examination not serious enough, and therefore show not their maximum performance. The low number of stations, which are conducted by the students, might be another source of bias of the individual results with respect to objective performance. Chance plays an important role, if previous experience from “practice” with the addressed differential diagnoses is present, or if a specific skill is needed which has been taught only in one lecture. Furthermore success or failure at the first station might influence the future performance and for sure the length of time from lecture to examination has an influence on the result. A restriction with respect to measuring performance is the fact that not all pathologic findings can be simulated well (i.e. blood pressure and heart rate) and the change of standardised patients and simulators pose problems for accomplishing the task by the students. In spite of this potential bias concerning measurement of individual performance and quality of test, the authors share the opinion, that the results altogether can be used as feedback to the faculty. In this sense the OSCE decision making in its present form is not a reliable instrument for measurement of performance, but an instrument for reflexion of individual and discipline specific - and moreover by the spectrum of 18 clinical disciplines also faculty-related – teaching related strengths and weaknesses and is therefore able to contribute to the “learning success” of a faculty.

## 5. Conclusions

The OSCE decision making has been worked up to an important feedback tool in the Marburg course of studies in human medicine. The fact, that it is interdisciplinary is for many disciplines a chance to participate, because they do not have to fill several stations from a certain discipline, but they can assess a part of their learning objectives. At the same time the totality of stations tests the majority of practical skills defined in the curriculum, and a lot of competence oriented learning targets are evaluated, which so far are not addressed in any other examination. These results encourage to present regularly a summary of analysed examination data to the disciplines and to stimulate the discussion of their specific results.

Being one of the few competence oriented examinations at the faculty of medicine in Marburg, it is desirable to spread this format to more stations. Within the planned restructuring of the curriculum and the future consideration of reimbursement of educational trained medical examiners this possibility seems to come true in practice. In addition, it makes sense and it is feasible, to consult and to supervise students, who performed unsatisfactory in this examination during their further course of studies and their practical year. In one case this has already been done.

## Competing interests

The authors declare that they have no competing interests.

## Figures and Tables

**Table 1:**
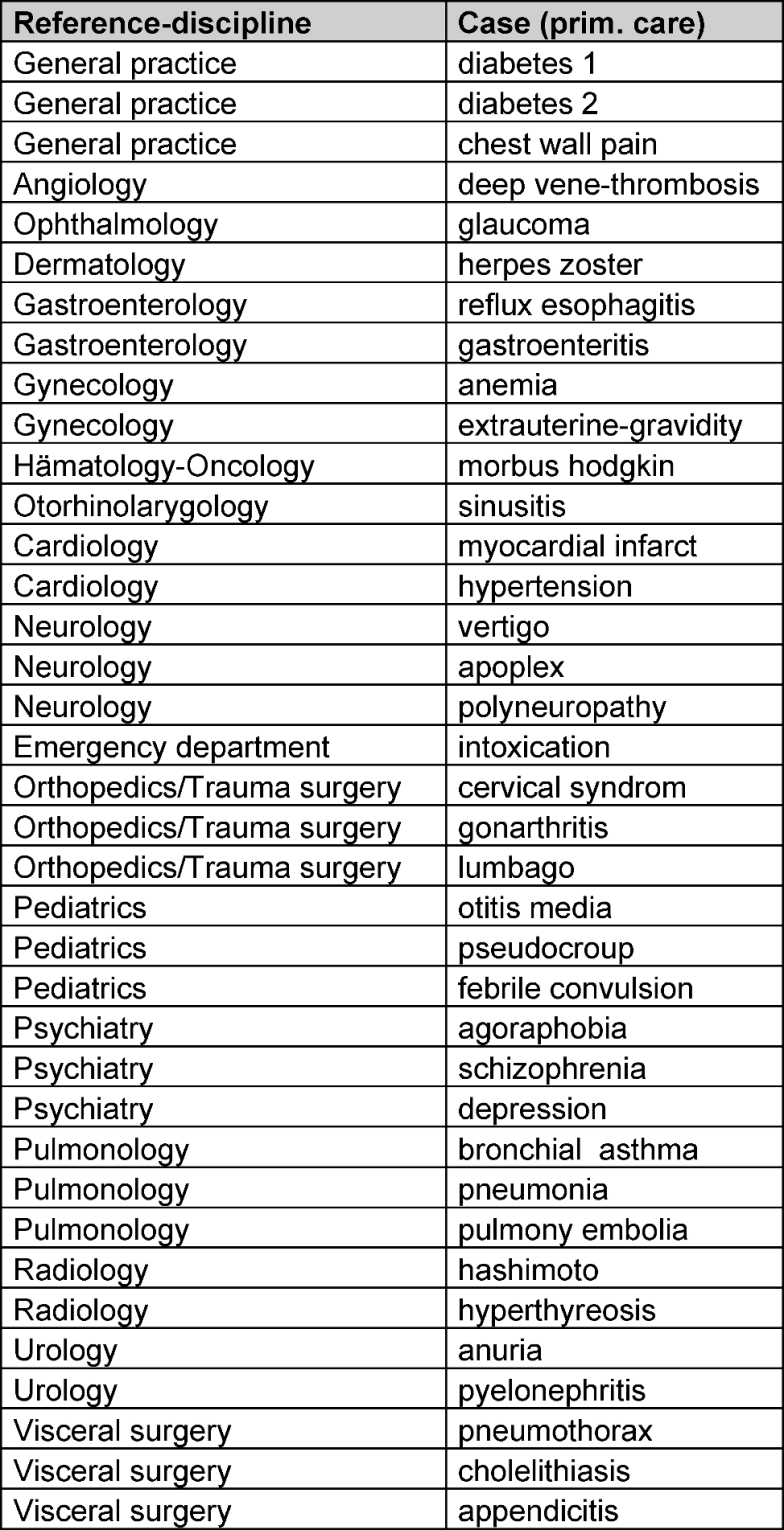
Reference disciplines (N=18) and corresponding cases/stations (N=37) for “OSCE decision making“ during the teaching year 2014/2015

**Table 2:**
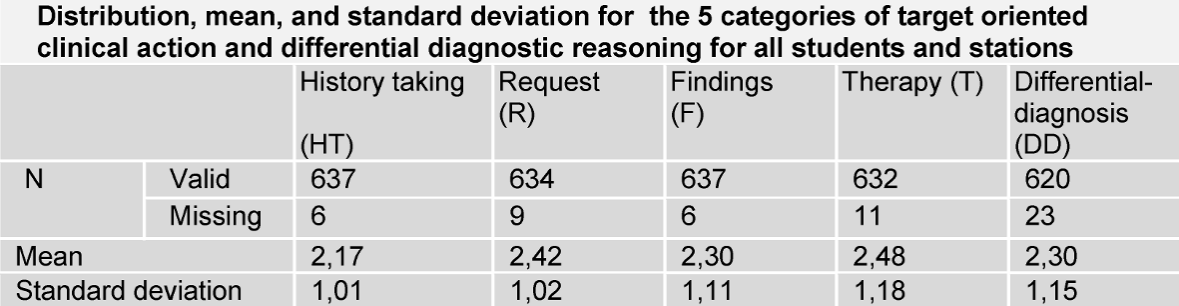
Distribution, Mean and Standard deviation for the 5 categories of target oriented clinical action for all students (N=218) and stations (N=37)
